# Time to Include Fine Specificity Anti‐Citrullinated Protein Antibodies in the Routine Diagnosis and Management of Rheumatoid Arthritis?

**DOI:** 10.1002/art.40767

**Published:** 2019-02-09

**Authors:** Anja Schwenzer, Anne‐Marie Quirke, Anna B. Montgomery, Patrick J. Venables, Harlan R. Sayles, Wolfgang Schlumberger, Jeffrey B. Payne, Ted R. Mikuls, Kim S. Midwood

**Affiliations:** ^1^ Kennedy Institute of Rheumatology Oxford UK; ^2^ University of Nebraska Medical Center Omaha NE; ^3^ Euroimmun Medizinische Labordiagnostika AG Lübeck Germany; ^4^ University of Nebraska Medical Center and Veterans Affairs Nebraska–Western Iowa Health Care System Omaha NE

Antibodies to citrullinated protein antigens (ACPAs) are part of the classification criteria for rheumatoid arthritis (RA) 1. ACPAs can be measured using generic tests, such as the cyclic citrullinated peptide (CCP) assays, that use artificially generated synthetic peptides with no homology to known proteins, which provide good diagnostic sensitivity and specificity. Peptides from real autoantigens (fine specificities) tend to be of lower diagnostic sensitivity and are used primarily for research into disease pathogenesis, rather than clinical management. In a recent study 2, we found 2 groups of ACPA fine specificities associated with different RA risk factors. One, dominated by antibodies to citrullinated α‐enolase peptide 1 (CEP‐1), was linked to smoking and shared epitope as previously described 3. The second group, linked to antibodies to a citrullinated peptide from tenascin‐C (cTNC5), was associated with infection with the periodontal pathogen *Prevotella intermedia*. The relative distinctions between anti–CEP‐1 and anti‐cTNC5 prompted us to ask whether testing for these 2 fine specificities in combination with anti‐CCP2 would be clinically useful in improving diagnosis or identifying clinical subsets among patients with RA.

We reanalyzed previous data from 287 patients with RA and 330 osteoarthritis controls, and we retested all of the sera for anti–CEP‐1 using a commercially available anti–CEP‐1 enzyme‐linked immunosorbent assay (Euroimmun), optimized for clinical use. The diagnostic sensitivity with anti–CEP‐1 was increased from 39% 2 to 48%, with a specificity of 98%. We then examined whether the addition of anti–CEP‐1 and anti‐cTNC5 to CCP2 increased the overall diagnostic sensitivity. We identified 8 patients with RA who were negative for anti‐CCP2 but positive for either or both of the fine specificities (Figure 1A), increasing the overall diagnostic sensitivity with ACPA from 84.5% obtained with anti‐CCP2 alone to 87% if all 3 assays were combined. We predict that this modest increase in sensitivity could be an underestimate caused by ascertainment bias because anti‐CCP2 positivity may have influenced the recruitment of patients to this cohort. In previous studies, using cohorts in which many of the patients were recruited before the CCP tests became available, anti–CEP‐1 and/or anti‐cTNC5 positivity was higher in the anti‐CCP2–negative RA patients, increasing overall diagnostic sensitivity by ~5% 2, 3, 4. Therefore, the inclusion of these 2 additional fine specificities at the point of diagnosis could increase the sensitivity even further, though clearly this needs to be confirmed in prospective studies.

**Figure 1:**
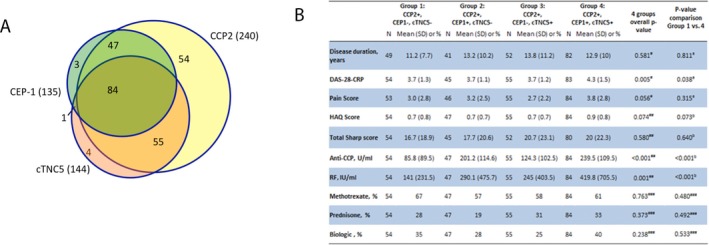
**A**, Venn diagram showing the overlap between citrullinated α‐enolase peptide 1 (CEP‐1), citrullinated tenascin‐C (cTNC5), and cyclic citrullinated peptide 2 (CCP2) antibody responses in 287 rheumatoid arthritis (RA) patients. For each anti–citrullinated protein antibody (ACPA), the total number of patients with positivity is shown in parentheses; overlaps in positivity are represented by numbers within the circles. Thirty‐six patients (12.5%) were negative for CEP‐1, cTNC5, and CCP2 antibodies (including 3 patients for whom no data on anti‐CCP2 were available). **B**, Disease characteristics of patients with RA according to positivity for different combinations of ACPA. # = by analysis of variance (ANOVA); ## = by Kruskal‐Wallis test; ### = by chi‐square test; a = ANOVA with post hoc pairwise comparisons; b = by Kruskal‐Wallis test with Dunn's test for pairwise comparisons. DAS28‐CRP = 28‐joint Disease Activity Score using the C‐reactive protein level; HAQ = Health Assessment Questionnaire; RF = rheumatoid factor.

We then investigated whether positivity for anti–CEP‐1 and/or anti‐cTNC5 identified distinct clinical subsets. For this analysis, we omitted the anti‐CCP2–negative patients, as numbers were too small for statistical evaluation. We identified no difference in joint disease between patients who were anti‐cTNC5 positive versus those who were anti–CEP‐1 positive. However, when both fine specificities were combined with anti‐CCP2, the 28‐joint Disease Activity Score (DAS28) 5 in patients positive for all 3 markers was significantly increased (*P* = 0.038) compared to that in patients positive for only CCP2, with the difference in the Health Assessment Questionnaire score 6 approaching significance (*P* = 0.073). Triple‐positive patients also had significantly higher levels of anti‐CCP2 (*P* < 0.001) and rheumatoid factor (*P* < 0.001) (Figure 1B). This increase in disease severity with multiple antibody specificities is perhaps not surprising, as a similar phenomenon has long been known to occur in systemic lupus erythematosus (SLE) 7.

In SLE, at least 6 different specificities would be routinely examined in every patient at presentation, with diagnosis established in the small proportion of antinuclear antibody–negative patients by the presence of anti‐Ro antibodies. The same antibody also identifies rare but important clinical subsets: for example, an association with congenital heart block in babies of anti‐Ro–positive mothers 7. In our patients with RA, no distinct clinical subsets were found, but we only examined joint‐specific variables. It is possible that associations with extraarticular features may be recognized in future studies. Indeed, while anti–citrullinated vimentin antibodies are linked with bone loss 8, anti–citrullinated fibrinogen antibodies have been associated with atherosclerosis 9. For anti–CEP‐1, a link with rheumatoid lung disease may be predicted by the association with smoking 3 and a high rate of positivity in patients with RA and bronchiectasis 10. Whether different ACPA fine specificities actively drive distinct aspects of joint disease and/or comorbidities also remains to be fully understood. We suggest that the only way to establish whether fine specificities are useful in improving diagnosis or identifying clinical subsets is to incorporate at least these 2 fine specificities into prospective studies so that in RA, multiple antibody assays become routine parts of clinical diagnosis and management.

*Supported by the European Community IMI (Innovative Medicines Initiative) project BTCure (grant 115142‐2), the Kennedy Trust for Rheumatology Research (grant AZRYXS00), and a Veterans Affairs Clinical Science Research and Development Merit Award (CX000896). Dr. Mikuls’ work was supported by a grant from the NIH/National Institute of General Medical Sciences (U54‐GM‐115458). Dr. Midwood's work was supported by an Arthritis Research UK Senior Fellowship (grant 20003). Drs. Schwenzer, Venables, and Midwood have filed patents related to the diagnostic utility of cTNC5. Dr. Midwood is founder of, and consultant to, Nascient Ltd. Drs. Schwenzer and Quirke contributed equally to this work*.

## Author contributions

All authors were involved in drafting the article or revising it critically for important intellectual content, and all authors approved the final version to be published. Dr. Midwood had full access to all of the data in the study and takes responsibility for the integrity of the data and the accuracy of the data analysis.

### Study conception and design.

Schwenzer, Quirke, Venables, Schlumberger, Payne, Mikuls, Midwood.

### Acquisition of data.

Schwenzer, Quirke, Montgomery, Venables, Payne, Mikuls, Midwood.

### Analysis and interpretation of data.

Schwenzer, Quirke, Venables, Sayles, Payne, Mikuls, Midwood.
